# Molecular and Physiological Effects of 17α-methyltestosterone on Sex Differentiation of Black Rockfish, *Sebastes schlegelii*

**DOI:** 10.3390/genes15050605

**Published:** 2024-05-09

**Authors:** Haijun Huang, Yuyan Liu, Qian Wang, Caichao Dong, Le Dong, Jingjing Zhang, Yu Yang, Xiancai Hao, Weijing Li, Ivana F. Rosa, Lucas B. Doretto, Xuebin Cao, Changwei Shao

**Affiliations:** 1 College of Fisheries and Life Science, Shanghai Ocean University, Shanghai 201306, China; huanghaijun0326@163.com; 2 State Key Laboratory of Mariculture Biobreeding and Sustainable Goods, Yellow Sea Fisheries Research Institute, Chinese Academy of Fishery Sciences, Qingdao 266071, China; liuyy@ysfri.ac.cn (Y.L.); wangqian2014@ysfri.ac.cn (Q.W.); bingo0635@163.com (C.D.); dongle164516@gmail.com (L.D.); jingjingzhang0307@163.com (J.Z.); yyu13156168974@163.com (Y.Y.); best_hxc@163.com (X.H.); lwj_mailbox_1@163.com (W.L.); lucas.doretto@unesp.br (L.B.D.); 3 Department of Structural and Functional Biology, Institute of Biosciences, São Paulo State University (UNESP), Botucatu 01049-010, Brazil; ivana.felipe@unesp.br; 4 School of Marine Sciences, Ningbo University, Ningbo 315211, China; caoxvebin1978@163.com; 5 Laboratory for Marine Fisheries Science and Food Production Processes, Qingdao Marine Science and Technology Center, Qingdao 266237, China

**Keywords:** *S. schlegelii*, 17α-methyltestosterone, gonadal development, sex reversal, sex hormone, transcriptomics

## Abstract

It is widely known that all-female fish production holds economic value for aquaculture. *Sebastes schlegelii*, a preeminent economic species, exhibits a sex dimorphism, with females surpassing males in growth. In this regard, achieving all-female black rockfish production could significantly enhance breeding profitability. In this study, we utilized the widely used male sex-regulating hormone, 17α-methyltestosterone (MT) at three different concentrations (20, 40, and 60 ppm), to produce pseudomales of *S. schlegelii* for subsequent all-female offspring breeding. Long-term MT administration severely inhibits the growth of *S. schlegelii*, while short term had no significant impact. Histological analysis confirmed sex reversal at all MT concentrations; however, both medium and higher MT concentrations impaired testis development. MT also influenced sex steroid hormone levels in pseudomales, suppressing E2 while increasing T and 11-KT levels. In addition, a transcriptome analysis revealed that MT down-regulated ovarian-related genes (*cyp19a1a* and *foxl2*) while up-regulating male-related genes (*amh*) in pseudomales. Furthermore, MT modulated the TGF-β signaling and steroid hormone biosynthesis pathways, indicating its crucial role in *S. schlegelii* sex differentiation. Therefore, the current study provides a method for achieving sexual reversal using MT in *S. schlegelii* and offers an initial insight into the underlying mechanism of sexual reversal in this species.

## 1. Introduction

Sex differentiation in fish is a complex developmental process influenced by the sex-determination mechanism, known as genetic sex determination (GSD) and environmental sex determination (ESD) [1]. Genetic and environmental sex determination (GSD + ESD) act sometimes in synergy to induce the male and female sex-differentiation process during the sensitive period of gonadal development [2]. Moreover, the development of gonad primordium is particularly sensitive to environmental factors, activating genes associated with sex differentiation and the synthesis of sex hormones, thereby determining the trajectory of sex differentiation [3,4].

Sex steroid hormones are considered the major inducers of gonadal sex differentiation and are also responsible for the maintenance of the differentiated gonad [5]. For instance, exogenous hormones, such as 4-chlorotestosterone, 17α-methyltestosterone (MT), and 17β-hydroxytrenbolone acetate, are commonly used to induce female androgenesis [6,7,8]. Conversely, the production of sex reversal toward the female phenotype is achieved with hormones such as estrone, estradiol, estriol, and diethylstilbesterol [9,10,11]. To date, MT is the most commonly used male sex-regulator hormone in a variety of fish [12]. More specifically, MT can efficiently reverse the biological sex of genetic females, producing XX males and promoting the development of male secondary sex characteristics [13].

*Sebastes schlegelii*, commonly known as blackfish or rockfish, inhabits the coastal waters of China, Korea, and Japan [14]. As a commercially valuable marine fish, its meat quality and nutrient density attract global attention. Moreover, *S. schlegelii* exhibits XX/XY sex determination, showcasing distinct sex dimorphism, with females surpassing males in growth [15]. In this sense, achieving all-female production could significantly enhance breeding profitability. Therefore, the current study aimed to induce XX *S. schlegelii* sex reversal using 17α-methyltestosterone before gonadal differentiation (20–68 days post-birthday). Further, the effects of MT exposure on mechanisms governing sex differentiation were investigated through histology, hormone level, and transcriptional expression analysis.

## 2. Materials and Methods

### 2.1. Fish Maintenance

Broodfish of *S. schlegelii* were reared and maintained at Muping Aquaculture Base (Yantai, Shandong Province) and all procedures were followed by the regulation of the Institutional Animal Care and Use Committee (IACUC) of the Yellow Sea Fisheries Research Institute (YSFRI) CAFS (Approval No. 2024010). Prior to steroid treatment, the *S. schlegelii* were temporarily reared in a 6 × 6 × 1.2 m nursery tank until reaching 19 days post-birthday (dpb). Subsequently, the individuals were randomly transferred to 75 L tanks (*n* = 600) within a seawater-filtered system under controlled photothermal conditions (18–26 °C).

### 2.2. Experimental Design and Steroid Treatment

Juvenile individuals at 20 dpb (*n* = 600) were randomly divided into four experimental groups: the control and three 17α-methyltestosterone (MT) (≥97%, CAS: 58-18-4, Aladdin, Shanghai, China) treatment groups with desired concentrations, namely low (20 ppm), medium (40 ppm), and high (60 ppm). For this purpose, MT was dissolved in 95% alcohol, followed by stock-solution preparation. Subsequently, the solution was sprayed onto the feed to create MT feed compounds. All MT-treated diets were dried and then stored at 4 °C before feeding. The MT feeding was administered from 20 to 68 dpb stage, and after that, the feeding of the fish continued on the control diet without the hormone. Three breeding-environment replicates of each treatment group were set up, and MT concentrations were based on previous studies [16,17,18]. After the 140 dpb stage period, body length, total length, and weight of each individual were measured, and fin, whole-body tissue, and gonadal tissue samples were collected for subsequent hormone-level analysis histological, sex determination, and transcriptomic analyses.

### 2.3. Genetic Sex Determination

To determine fish genotypic sex, fin samples of each animal were first dissected and preserved in ethanol. Subsequently, DNA extraction was carried out using the Marine Animal Tissue Genomic DNA Extraction Kit (TIANGEN, Beijing, China) following the manufacturer’s instructions. The DNA concentration and OD260/OD280 ratio were measured by NanoDrop2000 ultramicro spectrophotometer (Thermo, Waltham, MA, USA). In addition, DNA integrity was verified through 1.0% agarose electrophoresis. Subsequently, primers were designed to identify the genetic sex of all the samples (Supplementary Material Table S1) [15].

### 2.4. Histology

In order to evaluate the influence of MT exposure during the gonadal differentiation of *S*. *schlegelii*, first, the sex of each individual was determined through PCR. And, histological analysis was performed on *S*. *schlegelii* gonads at 140 dpb (*n* = 10–15/per group) for both the control group (XX and XY) and those subjected to MT treatments (XX) (20 ppm, 40 ppm, and 60 ppm). Briefly, the gonads were first collected and fixed overnight in 4% paraformaldehyde (Solarbio, Beijing, China). Posteriorly, the samples were dehydrated in alcohol (Sinopharm, Beijing, China) and xylene (BaSO) and embedded in paraffin wax (Leica, Wetzlar, LLH, Germany). Then, histological sections of 3 μm thickness were obtained and stained with hematoxylin (BaSO) and eosin (Sinopharm) for observation of general cellular structures under a microscope.

### 2.5. Sex Steroid Hormone Levels Following MT Exposure

In order to evaluate the influence of MT exposure during the sex differentiation of *S. schlegelii*, the levels of 17β-estradiol (E2), testosterone (T), and 11-keto testosterone (11-KT) were assessed using an enzyme-linked immunoassay (Competitive ELISA kit) (Standard, Qingdao, China). First, the sex of each individual was determined through PCR, and subsequent whole-body tissue homogenates of females, males, and pseudomales (MT 20 ppm) were conducted as described by [19]. The samples (*n* = 3/per group) were analyzed in duplicate, and the hormone levels were determined based on a standard curve.

### 2.6. RNA Extraction and cDNA Library Construction

Gonads from females, males, and pseudomales (MT 20 ppm) at 140 dpb stage (*n* = 3/per group) were collected and preserved in liquid nitrogen, followed by storage at −80 °C. Thereafter, total RNA extraction from the tissue samples was carried out using the Trizol method (Invitrogen, Carlsbad, CA, USA). The RNA concentration and OD260/OD280 ratio were determined using a NanoDrop2000 ultramicro spectrophotometer (Thermo Fisher Scientific, Waltham, MA, USA) for accurate detection of RNA integrity. Subsequently, qualified RNA samples were selected for the enrichment of mRNA and the construction of the cDNA library.

### 2.7. Transcriptome Sequencing and Data Analysis

The constructed libraries were initially quantified using a Qubit2.0 Fluorometer (Invitrogen); then, an Agilent 2100 bioanalyzer (Agilent, Santa Clara, CA, USA) was used to detect the insert size of the library. After passing the quality inspection, the machine sequencing was analyzed on the Illumina sequencing platform. The raw reads were processed for the removal of adapters and low-quality reads using FastQC [20]. The clean reads were mapped to the *S. schlegelii* genome (GCA014673565.1) using HISAT2 V2.0.5 [21]. FeatureCounts (1.5.0-p3) has been used to calculate the readings mapped to each gene [22], calculate fragments per kilobase million (FPKM) of each gene based on its length, and calculate the readings mapped to that gene. DESeq2 software (1.20.0) was used for differential expression analysis to screen differential expression genes (DEGs) [23]. The unigenes with *p-adj* < 0.05 and |log2(fold-change)| ≥ 1 were identified as significant DEGs. Gene ontology (GO) and Kyoto Encyclopedia of Genes and Genomes (KEGG) pathway enrichment analysis of differential genes were performed by clusterProfiler (3.8.1) [24].

### 2.8. Primer Synthesis and qRT-PCR Validation

Differentially expressed genes (DEGs) identified in the transcriptome analysis were chosen and subsequently validated through qRT-PCR. For this purpose, primers were designed using Primer Premier 5.0 software [25], and synthesized by Qingdao Ruibo Biotechnology Co., Ltd., Qingdao, China (Supplementary Material Table S1), primers reference [19,26,27]. The QuantiNova SYBR Green PCR Kit (Qiagen, Dusseldorf, NRW, Germany) was used for the qRT-PCR experiments. The qRT-PCR reaction was 10 µL, containing 2 × SYBR Green PCR Master Mix 5 µL, water 3.2 µL, cDNA 1 µL, and primer 0.4 µL. Cycles consisted of 95 °C for 2 min, followed by 40 cycles of 95 °C for 5 s and 60 °C for 10 s. Melting-curve reaction conditions were 95 °C for 15 s, 65 °C for 1 min, +0.11 °C/s to 95 °C, and 40 °C for 10 s, using a LightCycler ^®^ 480 II system (Roche, Basel, BS, Switzerland). *β-actin* was used as an internal reference gene and the relative mRNA levels of the selected genes were determined using the 2^−ΔΔCt^ method.

### 2.9. Growth and qRT-PCR Statistical Analysis

All data were represented by mean stand error (x ± SEM) and summarized using Microsoft Excel. Differences between groups were analyzed by One-Way (ANOVA) followed by Tukey’s multiple range test using Graphpad Prism 8.0.2 software [28]. All differences were considered statistically significant when *p* < 0.05.

## 3. Results

### 3.1. Effects of MT on the Growth of S. schlegelii

**Figure 1:**
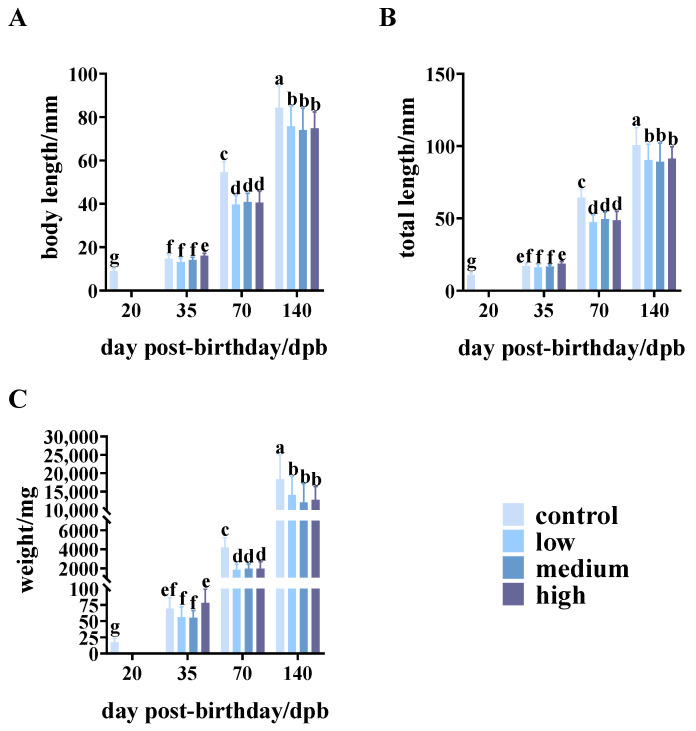
Growth of *S. schlegelii* exposed to 17α-methyltestosterone treatment and control group at 20, 35, 70, and 140 dpb stages. (**A**) Body length, (**B**) total body length, and (**C**) weight of *S. schlegelii*. Different letters indicate significant differences among groups (*p* < 0.05). Low: 20 ppm of MT, medium: 40 ppm of MT, high: 60 ppm of MT.

In the present study, we first analyzed the effects of different doses of 17α-methyltestosterone (MT) (20, 40, and 60 ppm) on the growth of *S. schlegelii* (Figure 1A). The results indicated that long-term administration of MT (70 dpb) significantly inhibits the growth of *S. schlegelii*, persisting until 72 days after withdrawal (140 dpb), whereas short-term exposure to low and medium MT concentrations (35 dpb) showed no discernible effect on growth (Figure 1A–C). At 70 and 140 dpb of development, significant decreases in body and total lengths of juvenile fish were observed in all MT concentrations compared to the control (Figure 1A,B). Similarly, a decreasing pattern was also observed in juvenile weight analyses at both 70 and 140 dpb stages (Figure 1C). Moreover, when analyzing the length, total length, and weight of the control animals, a significant increase in all parameters was observed across all development stages, with particularly pronounced growth at 140 dpb compared to the other groups.

### 3.2. Effects of MT on Gonad Differentiation and Development of S. schlegelii

**Figure 2:**
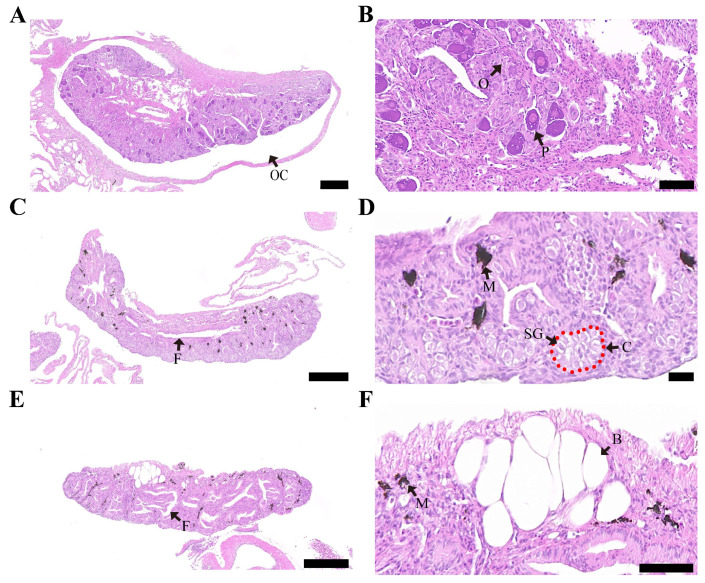
Histological changes in gonads of *S. schlegelii* in 140 dpb. (**A**) control ovary, scale bars = 200 μm; (**B**) control ovary, scale bars = 50 μm; (**C**) control testis, scale bars = 200 μm; (**D**) control testis, scale bars = 50 μm; (**E**) abnormal testis (MT 40 ppm), scale bars = 200 μm; (**F**) abnormal testis (MT 40 ppm), scale bars = 50 μm. OC: ovarian cavity; O: oogonia; P: oocyte at the perinucleolus stage; F: fissure structure; C: cysts; M: melanophore; SG: spermatogonia; B: bubble-like structure.

Histological results showed that the control females (XX) developed a typical ovarian cavity structure (Figure 2A). At the cytological level, oogonia and oocytes at the perinucleolus stage were observed (Figure 2B). The control males (XY) exhibited a fissure structure (Figure 2C), while melanocytes and spermatogonia were also observed. Spermatogonia were located within cysts (Figure 2D). In contrast, all females (XX) treated with MT displayed female-to-male sex reversal (pseudomales). However, as MT treatment concentrations increased, a certain proportion of abnormal testicles developed in the genetic females (Figure 2E), though the overall structure resembled that of the testicle. Curiously, a bubble-like structure was observed, which was only observed in the middle and high concentrations of the MT treatment (40 and 60 ppm) (Figure 2F).

Histological assessment of the gonads revealed that male and female control individuals developed typical testicles and ovaries (Table 1). In contrast, treatments with MT at low (20 ppm), medium (40 ppm), and high (60 ppm) concentrations resulted in 100% sex reversal in all groups, alongside the development of normal testicles in female (XX) individuals at rates of 100%, 92.9%, and 81.8%, respectively. Furthermore, the percentage of abnormal testicular development showed a gradual increase corresponding to the concentration of MT. In conclusion, the low-concentration group exhibited the most effective treatment, and females (XX) from this group were used as pseudomale subjects for subsequent analysis.

### 3.3. Analysis of Sex Steroid Hormone Levels

In order to explore the mechanism of sex reversal of the pseudomale in the low-concentration group, we additionally analyzed the sex steroid levels in pseudomales subjected to low-concentration MT treatment, comparing them to those of normal females and males.

**Figure 3:**
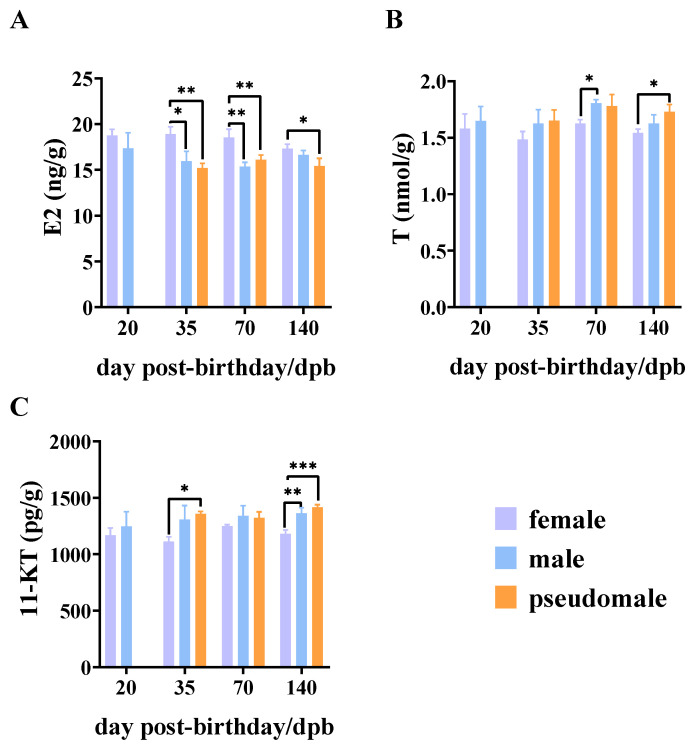
Effects of 17α-methyltestosterone (low-dose 20 ppm) on sex steroid hormones in *S. schlegelii* during sex differentiation stages (**A**) E2 levels (**B**) T levels, and (**C**) 11-KT levels. Asterisks denote statistically significant differences (* *p* < 0.05; ** *p* < 0.01; *** *p* < 0.001).

The findings revealed a significant impact of low-concentration MT treatment on the levels of E2, T, and 11-KT in both female and male individuals, as well as pseudomales (Figure 3A–C). In detail, females displayed significantly higher E2 levels than males at both the 35 and 70 dpb stages, while males and pseudomales showed a decrease in E2 levels (Figure 3A). At 140 dpb, only pseudomales showed significant differences compared to females (Figure 3A). Regarding T levels, males at 70 dpb and pseudomales at 140 dpb exhibited elevated T levels when contrasted with females (Figure 3B). Moreover, elevated levels of 11-KT were noted in males at 140 dpb and in pseudomales at both the 35 and 140 dpb stages (Figure 3C). In summary, our results indicate that MT had a significant effect on sex steroid hormone levels in pseudomales, suppressing E2 levels while increasing T and 11-KT levels.

### 3.4. Transcriptome Sequencing Output and Data Quality Control

Transcriptome sequencing was conducted on gonad samples from females, males, and pseudomales, followed by a quality assessment. In the current study, a total of 57.69 Gb of clean data were generated from 60.43 Gb of raw data. After filtering, the Q20 and Q30 data were above 97.81 and 94.21%, respectively, and the total average of the GC base was 50.53% for females, 48.96% for males, and 48.65% for pseudomales (Supplementary Material Table S2). These results indicate the high quality of sequencing data, establishing its suitability for subsequent analysis.

**Figure 4:**
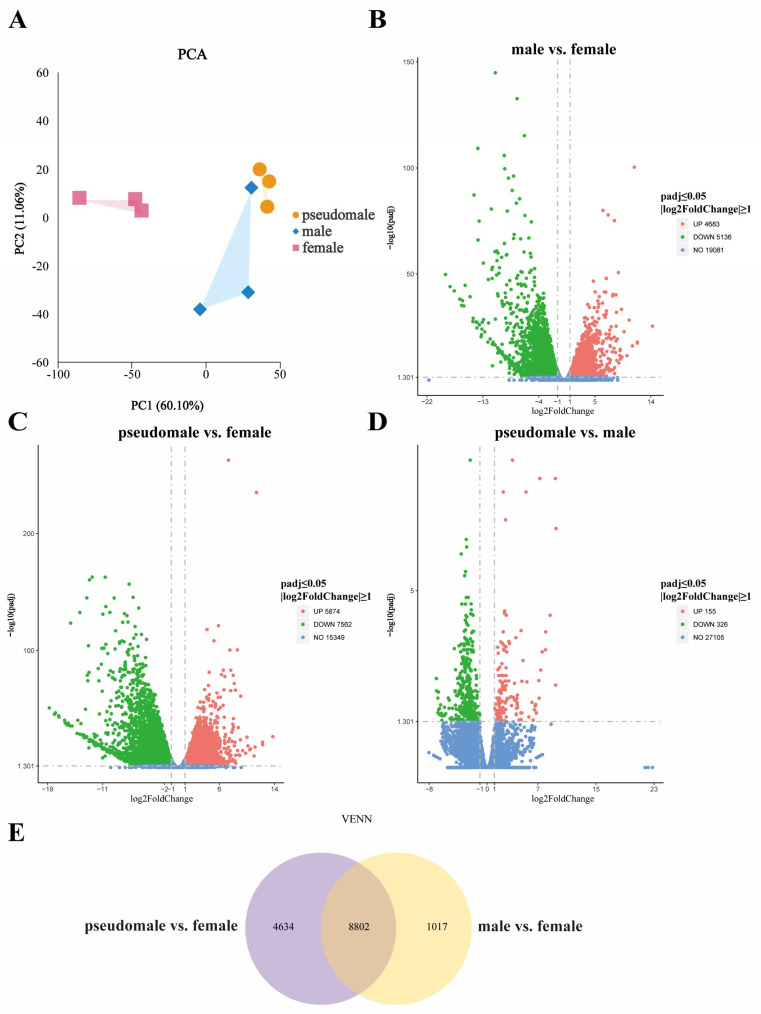
Principal component analysis and DEGs distribution among females, males, and pseudomales. (**A**) Principal component analysis (PCA). (**B**–**D**) Volcano plots show the numbers and distribution of DEGs in male vs. female, pseudomale vs. female, and pseudomale vs. male, respectively. (**E**) Venn mainly shows the numbers and distribution of DEGs in pseudomale vs. female and male vs. female.

Based on the FPKM values of all genes in females, males, and pseudomales, a PCA analysis was conducted. The PCA analysis revealed that the three experimental groups were distinctly differentiated, indicating that this data could be used for subsequent analysis (Figure 4A). In addition, the PCA results indicate a clear distinction between females and males, with pseudomales showing a greater overall similarity to males.

According to the differentially expressed gene analysis, 9819 DEGs were identified in males compared to females, with 4683 being up-regulated and 5136 down-regulated (Figure 4B). Regarding the 13,436 DEGS identified between pseudomales and females, 5874 were up-regulated and 7562 were down-regulated (Figure 4C). Furthermore, only 481 DEGs in pseudomales compared to males were identified, 155 of which were up-regulated and 326 down-regulated (Figure 4D). According to the results of the DEGs pairwise comparison between females, males, and pseudomales, the pseudomales showed an mRNA expression pattern more resembling males than females. To further screen the sex-regulating DEGs, a Venn analysis was performed between all groups (Figure 4E).

Our results identified 4634 DEGs between pseudomales and females (exclude those shared by males), and 1017 DEGs in males compared to females (exclude those shared by pseudomales). Moreover, 8802 DEGs were shared among all groups analyzed.

### 3.5. Functional Enrichment of DEGs

To further explore the biological mechanism functions of MT-induced sex reversal in *S. schlegelii*, 8802 shared DEGs anteriorly identified through Venn analysis were further screened. Among them, 4067 and 4732 DEGs were co-up-regulated and co-down-regulated in pseudomales and males, respectively, compared with females. Then, these co-up-regulated (co-up) and co-down-regulated (co-down) DEGs were enriched by GO and KEGG analysis.

GO analysis showed that 867 and 996 DEGs were successfully annotated in co-up and co-down, respectively (Supplementary Material Figure S1A,B). In co-up DEGs, the biological process (BP) functions most enriched were small GTPase-mediated signal transduction, regulation of signal transduction, and regulation of cell communication. Regarding the cellular components (CC) identified, ribosomes, ribonucleoprotein complexes, and extracellular matrix were the most enriched. Further, structural molecule activity, structural constituents of ribosomes, and transmembrane receptor protein kinase activity were the GO terms observed in molecular function (MF) (Supplementary Material Figure S1A). In co-down DEGs, the functions related to BP include RNA processing, ncRNA metabolic process, and cellular response to DNA damage stimulation. In the CC, non-nuclear portions, membrane-enclosed lumen, and organelle lumen were identified, and catalytic activities, acting on RNA, RNA binding, and nucleotidyltransferase activity were observed in molecular function (MF) (Supplementary Material Figure S1B). It is noteworthy that the GO term related to cell cycle and meiosis were also enriched in co-down (Supplementary Material Table S3).

**Figure 5:**
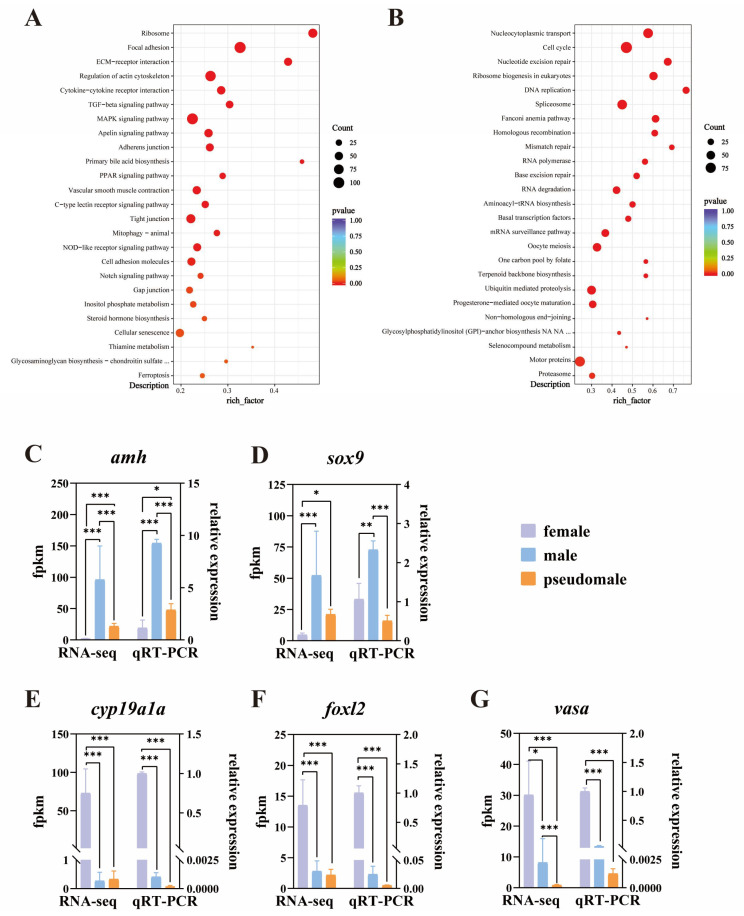
KEGG enrichment analysis of up-regulated (co-up) and downregulated (co-down) DEGs followed by RT-qPCR validation. (**A**) KEGG of DEGs co-up by pseudomale vs. female and male vs. female. (**B**) KEGG of DEGs co-down by pseudomale vs. female and male vs. female. Enrichment according to *p*-value < 0.05 threshold values. (**C**–**G**) Validation of RNA-seq results by qRT-PCR using 5 selected DEGs. Enrichment according to *p*-value < 0.05 threshold values. Asterisks indicated statistically significant differences (* *p-adj* < 0.05; ** *p-adj* < 0.01; *** *p-adj* < 0.001; * *p* < 0.05; ** *p* < 0.01; *** *p* < 0.001).

Based on the KEGG database, 148 and 159 pathways were enriched in co-up and co-down, respectively (Figure 5A,B). In the KEGG enrichment analysis, co-up DEGs were mainly involved in the ribosome, focal adhesion, ECM–receptor interaction, and regulation of the actin cytoskeleton. Moreover, pathways involved in sex differentiation, such as TGF-β signaling and steroid hormone biosynthesis, were also identified (Figure 5A). We also find co-down pathways mainly involved with nucleocytoplasmic transport, cell cycle, nucleotide excision repair, ribosome biogenesis in eukaryotes, and DNA replication. In addition, some pathways related to oocyte differentiation, such as oocyte meiosis and progesterone-mediated oocyte maturation, were also identified (Figure 5B).

Five sex-related DEGs were selected and validated using qRT-PCR to confirm the accuracy and reliability of the RNA-Seq data. The relative expression levels of *amh*, *sox9*, *cyp19a1a*, *foxl2,* and *vasa* obtained from qRT-PCR were consistent with the RNA-Seq results (Figure 5C–G). The results showed that male-related genes (*amh* and *sox9*) were highly expressed in males (Figure 5C,D). Ovarian-differentiation genes (*cyp19a1a* and *foxl2*) were highly expressed in females. MT significantly inhibited the *cyp19a1a* and *foxl2* expression levels in pseudomales (Figure 5E,F). Further, the germ cell marker (*vasa*) showed high mRNA expression in females in relation to the other groups (Figure 5G). In summary, the pseudomales showed a pattern of mRNA expression more resembling males than females.

## 4. Discussion

Recent studies have confirmed that MT not only induces sexual reversal in phenotypic females but rather exerts specific influences on the growth and development of juvenile fish [13]. For example, high concentrations of MT lead to a significant growth delay and adverse effects on gonad development in *Nibea albiflora* [29]. Moreover, the exposure of *Oreochromis spilurus* to MT results in severe growth inhibition at high concentrations, while low concentrations actively promote growth [30]. Consistent with these observations, our findings demonstrated that higher MT concentration at 35 dpb positively influences juvenile fish growth. Nevertheless, as the treatment duration extended, the growth rates in each concentration group significantly decreased in comparison to the control group. In addition, when comparing the body length, total length, and body weight of juvenile fish at 70 and 140 dpb, no significant differences were observed between different MT concentration groups; however, a decrease in growth parameters was observed in relation to the control. In summary, our results confirm that MT short-term exposure until 35 dpb can promote the growth of *S. schlegelii* juvenile fish; however, long-term treatment until 140 dpb significantly inhibits *S. schlegelii* juvenile growth.

In fish species, sex steroid hormones play an important role during the sensitive period of sex differentiation [5]. Several studies have confirmed that artificial hormone exposure in *N. albiflora* influences the expression of sex differentiation genes, consequently affecting gonad differentiation. Specifically, the treatment with MT up-regulates the expression of *dmrt1,* an important gene related to male sex differentiation, thereby inducing XX female-to-male sex reversal in *N. albiflora* [29]. It is widely known that the difficulty of hormone-induced sex reversal in juvenile fish lies in the control of treatment exposure and concentration. Prolonged treatment periods or high concentrations might, consequently, disrupt normal gonadal development, potentially leading to impaired gonadal maturation [29,31]. For example, induction of high E2 concentrations inhibits the expression of *vldr* and *fshr* genes in *Takifugu rufugu*, resulting in the inhibition of oocyte growth and division, thereby reducing the number of oocytes [31]. Additionally, prolonged exposure to E2 led to abnormal development in the ovaries of *Oryzias latipes* pseudofemales [32]. These findings suggest that regardless of the hormone used for inducing fish sex reversal, both the concentration and timing need to be carefully controlled. The current study showed that three doses of MT treatment successfully induced sex reversal in *S. schlegelii* juvenile fish, resulting in the development of testicles in XX individuals. Nevertheless, medium and high concentrations promote severe alterations in testis development, such as abnormal testicular development. In summary, the results show that the treatment effect in the low-concentration group (20 ppm) is the best concentration in this experiment, and future studies should adjust the treatment time and concentration according to the conclusion.

E2, T, and 11-KT are the most typical sex steroid hormones in fish, playing crucial roles in sex differentiation, gonad development, and reproduction. The interplay among these hormones, with T being aromatized into E2 under aromatase action and synthesizing 11-KT, establishes a mutual regulatory mechanism during the critical period of gonadal differentiation [33]. To date, researchers currently propose three mechanisms for inducing sexual reversal in female fish through MT. One of these mechanisms involves direct action on the gonad or hypothalamic–pituitary axis, influencing female androgen levels through feedback regulation, thus controlling the proliferation and differentiation of primordial germ cells [13]. Another mechanism involves reducing E2 levels by suppressing aromatase mRNA expression in juvenile fish, thus affecting ovary formation and achieving sex reversal [34]. Moreover, some studies suggest that MT primarily acts on the gonads, achieving sex reversal by regulating T levels. For example, a significant increase in T levels was found in *Siniperca chuatsi*, possibly attributed to MT inhibiting the conversion of T to E2 [35]. However, studies in *Gadus morhua* have shown that MT treatment does not significantly affect T levels [36]. Meanwhile, other research suggests that 11-KT is the most effective androgen in fish gonad development [37]. In a study of the Pacific halibut *Hippoglossus stenolepis*, 11-KT levels were significantly higher than T levels at all stages of testicular development [38]. Hence, regardless of the specific regulatory pathway through which MT induces sex reversal, the levels of E2, T, and 11-KT play pivotal roles in testicular development. Furthermore, in *S. schlegelii*, studies have shown that ovarian and testicular differentiation typically occurs around 35 dpb and 70 dpb, with the formation of the ovarian lumen and vas deferens, respectively [19]. Moreover, our study showed that, at 140 dpb, female germ cells have differentiated oocytes at the perinucleolus stage. In this study, it was found that E2 levels in females increased before 35 dpb and then gradually decreased, indicating a correlation between the increase in E2 levels and ovarian differentiation. Regarding male hormone levels, there was a gradual decrease in T levels before 35 dpb, followed by an increase in the 70 dpb stage, indicating that increased T levels could be associated with the promotion of testis differentiation. Furthermore, 11-KT levels in males consistently increased throughout the differentiation periods, reaching the highest level at 140 dpb, possibly due to delayed conversion of T to 11-KT levels. By comparing females, males, and pseudomales, it can be observed that, under the influence of MT, the E2 levels of pseudomales were significantly lower than those of normal females at 35 dpb. Thus, MT significantly inhibited E2 levels in females, inhibiting gonad development towards the ovary during rapid ovary differentiation. This result is consistent with the observed development of pseudomales induced by MT in *Paralichthys olivaceus* [34]. Moreover, pseudomales and males displayed elevated levels of T and 11-KT compared to females along the sex differentiation periods. Concerning 11-KT levels, our findings suggest that 11-KT levels are sensitive to MT treatment, and this may be due to the hypothalamic–pituitary axis effect, which enhances the conversion of T into 11-KT in fish, leading to increased 11-KT content through feedback regulation.

Despite the widespread application of hormones for sex control in diverse fish species, the molecular mechanism underlying the sex reversal of XX *S. schlegelii* remains poorly understood. Hence, to further explore the biological mechanism of MT-induced sex reversal in *S. schlegelii*, a transcriptome analysis was performed in pseudomales, normal females, and normal males exposed to low-dose MT.

We first performed a Venn analysis, and the 4634 DEGs in pseudomale vs. female may be mainly related to the abnormal testicular development of pseudomales caused by MT. The 1017 DEGs in male vs. female may be mainly involved in testicular and ovarian differentiation. The 8802 DEGs shared by pseudomale vs. female and male vs. female may explain the sex reversal in females, which will be the focus of further research. In this study, male-related genes (*amh* and *sox9*) were highly expressed in males; MT up-regulates the expression of *amh* in pseudomales. In agreement with these results, the enrichment analysis indicated that sex reversal in *S. schlegelii* may be closely related to the TGF-β signaling pathway and steroid hormone biosynthesis. As a representative member of the TGF-β signaling pathway, *amh* plays a key role in germ-cell proliferation, spermatogenesis, and male sex determination in fish [39,40,41,42,43]. In addition, *amh* shows a typical sex dimorphism in most fish, with a significantly higher expression in the testis than in the ovaries, compatible with a role in fish gonad differentiation and male gonad maintenance [44]. Further, it has been found that E2 treatment significantly inhibits *amh* expression of *Odontesthes bonariensis*, leading to the suppression of division and differentiation of testicular germ cells, and ultimately resulting in testicular degeneration [45]. Moreover, in *Danio rerio* studies, MT significantly up-regulates *amh* expression, accelerating gonad androgynism [46]. As the upstream regulator of *amh*, *sox9* also plays an important role in testis differentiation, and their expression is known to be positively correlated [47]. Similarly, in the current study, the expression levels of *amh* and *sox9* in females, males, and pseudomales followed the same trend at 140 dpb, with pseudomales and females showing significantly lower expression than males. Although MT did not affect the expression of *sox9*, *amh* expression was higher in pseudomales than in females. This result suggests that MT has a certain effect on the *amh–sox9* pathway, mainly acting on *amh* regulation.

Steroid hormone biosynthesis extensively regulates the level of sex hormones in fish, and, therefore, it is crucial in the process of sex differentiation [48]. Aromatase is a member of the cytochrome P450 family that converts androgens into estrogen [49]. Ovoviviparous fish have two aromatase genes, such as *cyp19a1a* and *cyp19a1b*, with *cyp19a1a* potentially influencing the trajectory of gonadal differentiation through the regulation of E2 levels [50]. In the current study, the expression levels of *cyp19a1a* showed a typical sex dimorphism, with a higher expression in the ovaries compared to the testis, as commonly observed in most fish species [26]. It is found that *foxl2* is involved in the transcriptional regulation of *cyp19a1a*. Therefore, during the early stages of gonad development, *foxl2* also influences ovary development and function in vertebrates [51,52]. Moreover, our study showed that *cyp19a1a* and *foxl2* were downregulated in males and pseudomales, consistent with another study of *Scophthalmus maximus* [53]. MT treatment significantly inhibited the expression levels of *cyp19a1a* and *foxl2* in pseudomales. In this sense, these findings suggest a correlation between sex-related genes and steroid hormones in MT-treated pseudomales, modulating the sexual differentiation of *S. schlegelii*. This is evidenced by the decrease in both the E2 levels and mRNA of *cyp19a1a* and *foxl2* and an increase in T and 11-KT levels.

Our study also analyzed *vasa* gene expression, mainly involved in the migration and differentiation of primordium germ cells in vertebrates, including in teleost [54,55,56]. In males, *vasa* expression was significantly lower than in females at 140 dpb, aligning with histological observations, indicating a period of accelerated ovarian development in females. In addition, MT significantly down-regulates vasa expression in pseudomales, showing a pattern of mRNA expression more resembling males than females. Furthermore, these findings might be associated with the down-regulation of pathways related to the meiotic cell cycle, oocyte meiosis, and maturation, as indicated by transcriptome analysis.

## 5. Conclusions

In the current study, the administration of the lowest MT dose (20 ppm) resulted in a complete sexual reversal of XX *S. schlegelii*, yielding pseudomales with 100% efficacy. This sex reversal was accompanied by the inhibition of E2 and an elevation in T and 11-KT levels. Through gonadal transcriptomic analysis, pseudomales exhibited gene-expression patterns more closely resembling males than females. In addition, the TGF-β signaling pathway and steroid hormone biosynthesis were identified as pivotal players in the *S. schlegelii* sex reversal process. Therefore, this study not only outlines a new method for achieving *S. schlegelii* female-to-male sex reversal but also provides initial insights into sexual reversal regulation in this species.

## Figures and Tables

**Table 1:** The development of *S. schlegelii* gonads.

Treatment /Genetic Sex	Sample/n	Ovary /Proportion ^1^	Normal Testis /Proportion ^2^	Abnormal Testis /Proportion ^3^
control/♀	15	15/100.0%	/	/
control/♂	12	/	12/100.0%	/
low/♀	12	/	12/100.0%	/
medium/♀	14	/	13/92.9%	1/7.1%
high/♀	11	/	9/81.8%	2/18.2%

♀: XX female. ♂: XY male. ^1^ Ovary/proportion: Number of samples with typical ovarian structure and its proportion within the group. ^2^ Normal testis/proportion: Number of samples with typical testis structure and its proportion within the group. ^3^ Abnormal testis/proportion: number of samples with typical testis structure and its proportion within the group.

## Data Availability

Data are contained within the article. RNA-seq data are submitted in NCBI (BioProject: PRJNA1085477).

## Supplementary Materials

The following supporting information can be downloaded at: https://www.mdpi.com/article/10.3390/genes15050605/s1, Table S1: Primers used in this study; Table S2: Sequencing data statistics; Table S3: GO term related to cell cycle and meiosis enriched by co-down-regulated DEGs; Figure S1: GO enrichment analysis of up-regulated (co-up) and down-regulated (co-down) DEGs followed. (**A**) GO of DEGs co-up by pseudomale vs. female and male vs. female. (**B**) GO of DEGs co-down by pseudomale vs. female and male vs. female.
