# Supplementary material for: Molecular and Physiological Effects of 17α-methyltestosterone on Sex Differentiation of Black Rockfish, Sebastes schlegelii
Source: Genes (Basel). 2024 May 9;15(5):605. doi: 10.3390/genes15050605 (PMC11120931; doi:10.3390/genes15050605)
Supplement: Supplementary file 1 [file genes-15-00605-s001.zip › genes-2947787-supplementary.pdf]

Supplementary Material

Table S1. Primers used in this study

Primers	Sequence (5'→ 3')	Usage
Sex-marker-F	GTAAACCAAGAACTGAGGAGGAG	Genetic sex determination
Sex-marker-R	GAGAAAAGCAGAAGTGGAATCA	
amh-F	AGCCTCACCGTGTCCCTA	qRT-PCR
amh-R	TCCCGTGTTCGTTCAAGTC	
sox9-F	AGAGGCTCCTTCCATCCG	qRT-PCR
sox9-R	AGCACGCCAGACTCAGAC	
cyp19a1a-F	CGCTGCACCGTAGTCGACAT	qRT-PCR
cyp19a1a-R	GCGATCACCATCTCCAACAC	
foxl2-F	CGACCAAGGAGAAAGAGCGA	qRT-PCR
foxl2-R	GCGATGAGAGCCACATAGGA	
vasa-F	GCAATGTGGTGTGTGGAACC	qRT-PCR
vasa-R	TCTGGCGGTTCTCTTTGGAC	
β-actin-F	CGGTATGGGCCAGAAAGACA	qRT-PCR
β-actin-R	GGTGACGATACCGTGCTCAA	

Table S2. Sequencing data statistics

Samples	Raw data/Gb	Clean data/Gb	Q20/%	Q30/%	GC/%	Total Map/%	Exon/%	Intron/%	Intergenic/%
female1	5.89	5.88	97.94	94.41	50.36	88.32	62.40	4.31	33.29
female2	6.40	6.39	98.04	94.65	50.04	88.06	61.75	4.60	33.66
female3	6.29	6.26	98.88	96.98	51.18	89.84	66.23	4.20	29.57
male1	7.56	6.99	98.00	94.59	48.83	90.82	52.87	4.97	42.16
male2	7.50	6.82	97.89	94.33	48.65	87.64	52.62	4.55	42.83
male3	6.72	6.13	97.84	94.21	49.40	87.88	53.28	4.85	41.87
pseudomale1	6.62	6.61	97.92	94.44	48.53	88.16	49.43	4.16	46.41
pseudomale2	7.05	6.64	97.85	94.30	48.53	89.59	50.88	5.42	43.71
pseudomale3	6.40	5.97	97.81	94.21	48.88	89.42	51.70	4.70	43.60

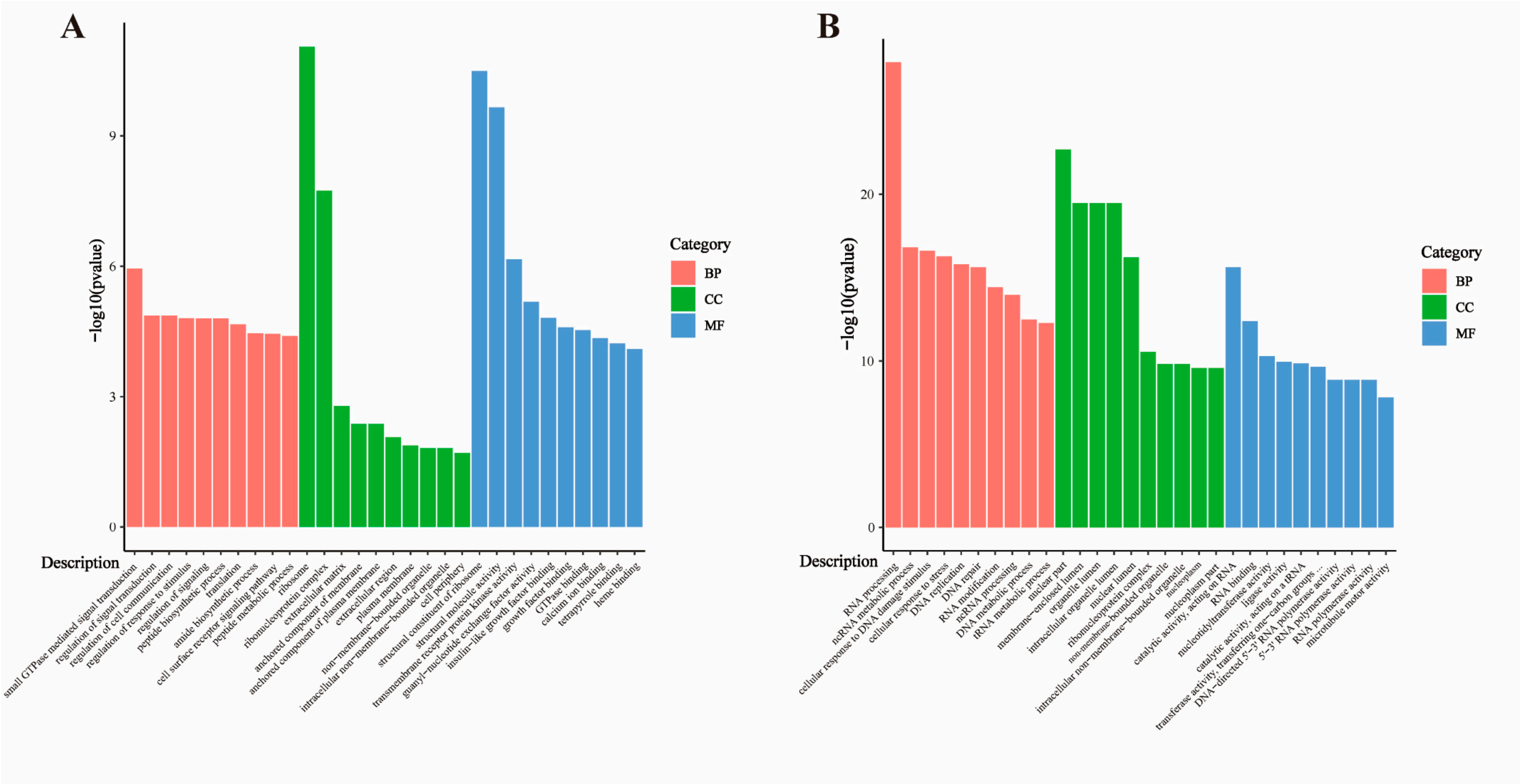

Figure S1. GO enrichment analysis of upregulated (co-up) and downregulated (co-down) DEGs followed. (**A**) GO of DEGs co-up by pseudomale vs. female and male vs. female. (**B**) GO of DEGs co-down by pseudomale vs. female and male vs. female.

Table S3. GO term related to cell cycle and meiosis enriched by co-down-regulated DEGs.

Category	GOID	Description	GeneRatio	BgRatio	pvalue	padj	Count
BP	GO:0007049	cell cycle	33/1260	58/7699	2.07E-12	9.14E-11	33
BP	GO:0022402	cell cycle process	22/1260	38/7699	6.68E-09	1.69E-07	22
BP	GO:0051321	meiotic cell cycle	9/1260	10/7699	7.02E-07	1.28E-05	9
BP	GO:0000278	mitotic cell cycle	11/1260	18/7699	2.25E-05	0.000290578	11
BP	GO:0051726	regulation of cell cycle	14/1260	32/7699	0.000233685	0.002223101	14
BP	GO:0010564	regulation of cell cycle process	7/1260	10/7699	0.000234894	0.002223101	7
BP	GO:0045786	negative regulation of cell cycle	10/1260	21/7699	0.000823647	0.006515417	10
BP	GO:1903047	mitotic cell cycle process	8/1260	15/7699	0.001096506	0.008546296	8
BP	GO:0000075	cell cycle checkpoint	7/1260	12/7699	0.001143334	0.008782131	7
